# Petroleum Hydrocarbon Contamination in Terrestrial Ecosystems—Fate and Microbial Responses

**DOI:** 10.3390/molecules24183400

**Published:** 2019-09-19

**Authors:** Adam Truskewycz, Taylor D. Gundry, Leadin S. Khudur, Adam Kolobaric, Mohamed Taha, Arturo Aburto-Medina, Andrew S. Ball, Esmaeil Shahsavari

**Affiliations:** 1 Centre for Environmental Sustainability and Remediation, School of Science, RMIT University, Bundoora, VIC 3083, Australia; adam.truskewycz@rmit.edu.au (A.T.); s3282982@student.rmit.edu.au (T.D.G.); leadin.khudur@rmit.edu.au (L.S.K.); adamkolobaric@gmail.com (A.K.); moohamedtaha@yahoo.com (M.T.); arturoaburto.medina@rmit.edu.au (A.A.-M.); andy.ball@rmit.edu.au (A.S.B.); 2 Department of Biochemistry, Faculty of Agriculture, Benha University, Moshtohor, Toukh, Qaliuobia 13736, Egypt

**Keywords:** petroleum hydrocarbon (PH), natural attenuation, bioremediation, microbial consortia

## Abstract

Petroleum hydrocarbons represent the most frequent environmental contaminant. The introduction of petroleum hydrocarbons into a pristine environment immediately changes the nature of that environment, resulting in reduced ecosystem functionality. Natural attenuation represents the single, most important biological process which removes petroleum hydrocarbons from the environment. It is a process where microorganisms present at the site degrade the organic contaminants without the input of external bioremediation enhancers (i.e., electron donors, electron acceptors, other microorganisms or nutrients). So successful is this natural attenuation process that in environmental biotechnology, bioremediation has developed steadily over the past 50 years based on this natural biodegradation process. Bioremediation is recognized as the most environmentally friendly remediation approach for the removal of petroleum hydrocarbons from an environment as it does not require intensive chemical, mechanical, and costly interventions. However, it is under-utilized as a commercial remediation strategy due to incomplete hydrocarbon catabolism and lengthy remediation times when compared with rival technologies. This review aims to describe the fate of petroleum hydrocarbons in the environment and discuss their interactions with abiotic and biotic components of the environment under both aerobic and anaerobic conditions. Furthermore, the mechanisms for dealing with petroleum hydrocarbon contamination in the environment will be examined. When petroleum hydrocarbons contaminate land, they start to interact with its surrounding, including physical (dispersion), physiochemical (evaporation, dissolution, sorption), chemical (photo-oxidation, auto-oxidation), and biological (plant and microbial catabolism of hydrocarbons) interactions. As microorganism (including bacteria and fungi) play an important role in the degradation of petroleum hydrocarbons, investigations into the microbial communities within contaminated soils is essential for any bioremediation project. This review highlights the fate of petroleum hydrocarbons in tertial environments, as well as the contributions of different microbial consortia for optimum petroleum hydrocarbon bioremediation potential. The impact of high-throughput metagenomic sequencing in determining the underlying degradation mechanisms is also discussed. This knowledge will aid the development of more efficient, cost-effective commercial bioremediation technologies.

## 1. Introduction

The introduction of petroleum hydrocarbons into a pristine environment immediately changes the nature of that environment. The introduced hydrocarbons kill or inhibit many microbial species, thereby altering the functionality of the microbial community and therefore the ecosystem [1]. Plants exposed to hydrocarbons are also affected by direct toxicity, prevention of access to light, and the inability to acquire nutrients and water due to oil restricting their movement through the soil matrix, all of which significantly impair plant productivity [2]. In the absence of functioning microbial biogeochemical networks and primary producers, the ability for the contaminated habitat to support higher-order life forms is limited.

When petroleum hydrocarbons enter the environment, the process of weathering occurs. Weathering of petroleum hydrocarbons in the environment involves physical (dispersion), physiochemical (evaporation, dissolution, sorption), chemical (photo-oxidation, auto-oxidation), and biological (plant and microbial catabolism of hydrocarbons) influences. The process of biodegradation, the focus of this review, is largely mediated through the natural soil microbial community. Natural attenuation represents the single, most important biodegradative process which removes petroleum hydrocarbons from the environment. It is a process where microorganisms present at the site degrade the organic contaminants without the input of external bioremediation enhancers (i.e., electron donors, electron acceptors, other microorganisms or nutrients) [3].

Microorganisms play a vital role in maintaining an ecologically balanced environment. They are responsible for regulating several processes in the soil ecosystem including recycling of nutrients, the decay of organic materials, and the formation of symbiotic relationships with plants [4]. Petroleum hydrocarbon contamination presents microorganisms with environments which have characteristics not usually experienced in natural conditions. Therefore, understanding the interaction of anthropogenic chemical pollutants within soil and their interactions with microbial communities is very important.

Microorganisms have varying mechanisms for adapting to and catabolizing petroleum hydrocarbons. Although individual microorganisms have mechanisms for hydrocarbon degradation (i.e., the enzyme-catalyzed breakdown of inorganic and organic pollutants) [5], other species may aid in this process by way of symbiotic relationships (i.e., release of glucose to aid in proliferation of hydrocarbon-degrading species or secretion of surfactants to render the oil more bioavailable) [6]. So successful is this natural attenuation process that an environmental biotechnology, bioremediation has developed steadily over the past 50 years based on this natural biodegradation process.

This review aims to describe the fate of petroleum hydrocarbons in the environment and discuss their interactions with abiotic and biotic components under both aerobic and anaerobic conditions. Furthermore, natural mechanisms for dealing with petroleum hydrocarbon contamination will be examined. Through a detailed examination of these processes, greater understanding of the potential of natural attenuation to remove all fractions of petroleum hydrocarbons will result, which can then be used to update sustainable and environmentally friendly bioremediation strategies used in commercial bioremediation.

The development of tailored microbial consortia for specific site conditions and different hydrocarbon classes is proposed. Furthermore, a monitored site remediation approach where differing microbial consortia are introduced at different stages of remediation may promote complete microbial hydrocarbon catabolism. Linking next-generation sequencing with the microbial functional analysis is also identified as an important prerequisite for understanding the capacity of microbial consortia to decontaminate petrogenic hydrocarbons at varying sites.

## 2. Oil Composition Changes upon Entering the Environment

Petrogenic hydrocarbons represent a complex group of pollutants that are usually released into the environment as a mixture consisting of n-alkanes, branched alkanes, cyclo-alkanes, and polyaromatic hydrocarbons (PAHs). These varying hydrocarbon compounds interact differently with their environment, often depending on their molecular weight and properties, with compounds varying in size and structure from C_1_ (methane) to n-C_40_+. Petroleum hydrocarbons can be divided into four categories based on their chemical structure—the saturates, the aromatics, the asphaltenes, and the resins [7].

**Figure 1:**
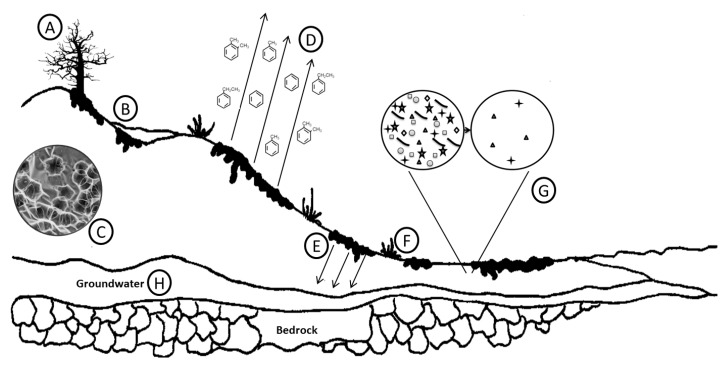
Fate of oil and site characteristic changes following a terrestrial oil spill event: (**A**) Plant death, (**B**) anaerobic zones, (**C**) altered soil structure, (**D**) volatilization, (**E**) hydrocarbon percolation, (**F**) aerobic zones, (**G**) initial decrease in microbial populations and diversity, (**H**) hydrocarbon contaminated groundwater.

Petroleum hydrocarbons in the environment undergo weathering, which may involve physical (dispersion), physiochemical (evaporation, dissolution, sorption), chemical (photo-oxidation, auto-oxidation), and biological (plant and microbial catabolism of hydrocarbons) influences [8] (Figure 1). Weathering may strongly impact the degradation potential of oil, and the degree and type of weathering will be different from site to site.

### 2.1. Volatilization

As the oil spreads over the landscape, the lighter fractions volatilize into the atmosphere. Different petroleum hydrocarbons may behave in different ways. Light crudes and gasoline may evaporate completely in warm conditions if they are not locked up in other environmental interactions, whereas bunker oil may only have volatilization rates of a few percent by weight [9]. Volatilization frees lighter aromatic (i.e., BTEX, and other simple ringed structures) from more complex oil mixtures (Figure 1). Saturated hydrocarbons with 1–5 ring structures are considered to be volatile or semi-volatile. Increasing temperature increases the rate and capacity of volatilization; however, increasing chain length reduces volatility [10]. Volatilization not only occurs on surface-dwelling oil, but it can also occur in the subsurface. Essaid et al. (2011) spent 25 years monitoring and modeling the fate of hydrocarbons from a burst crude oil pipeline in Bemidji, Minnesota, which released 1.7 million liters of oil into the environment. Oil which had percolated through the soil was still volatilizing and was creating a soil vapor plume in the saturated zone [11].

### 2.2. Dissolution

As a hydrocarbons chain length or number of aromatic rings increases, its solubility in water decreases. The dissolved concentration of petroleum hydrocarbons in water is reliant on the composition of the oil. A large quantity of viscous crude oil may have less dissolvable fractions than a smaller quantity of lighter-class oil. Dissolvable fractions at 20 °C generally possess hydrocarbon chains of below C_8_ [12]; however, the presence of polar nonhydrocarbon substances found within the environment can increase the solubility of C_34_^+^ n-alkane hydrocarbons in oils [13]. In addition, other light aromatic and polycyclic aromatic compounds (e.g., benzene, toluene, xylene, ethylbenzene (BTEX) and naphthalene) are commonly found within contaminated groundwater [14].

Salinity can have a marked effect on hydrocarbon solubility. In salinities of 350,000 ppm, up to 95% of all hydrocarbons can be displaced from solution. In addition, both temperature and sorption of hydrocarbons to soils can influence their ability to form a solution in water. Hydrocarbon solubility has shown to logarithmically decrease with the increasing boiling point of a hydrocarbon [13].

### 2.3. Sorption and Desorption

Many fractions of hydrophobic hydrocarbons have an affinity to sorb onto soil particles via several processes. These may include the partitioning of hydrocarbon into soil organic matter, diffusion into nanopores, which are inaccessible to microorganisms, or attachment and formation of strong bonds with sites on soils organic matter [15]. Soil structure and composition are likely to strongly influence sorption and desorption kinetics. Hydrocarbon sorption to soil has shown to increase with increasing organic matter, increasing clay content, and increasing hydrocarbon hydrophobicity. The recalcitrance of aged PAH fractions bound to soil components is increased due to their decreased water solubility and inaccessibility to microorganisms [16].

Desorbing strongly bound hydrocarbons from soil matrices has been achieved through the interaction between oil and bioactive compounds secreted from plants and microorganisms. In addition, plant roots can break apart aggregates and free bound hydrocarbons from nanopores [17]. During bioremediation of contaminated sites, a surfactant is often applied to reduce the viscosity of oil, promoting the release of oil from soil sorption [18]. Desorption of hydrocarbons from soil matrices is also increased with higher sand content, increased temperatures, and increased soil moisture [16].

## 3. Toxicity of Hydrocarbons to Microorganisms and Microbial Communities

Once released into the environment, certain petroleum hydrocarbon fractions exhibit high toxicity to soil biota and have been linked with inducing serious health problems in humans, including cancer [19]. Site contamination with petroleum hydrocarbons reduces the diversity and evenness of a microbial community [20] for a number of reasons including:

- direct toxicity of the chemical compounds,
- the locking away of nutrients and water, preventing microorganisms from obtaining essential building blocks for proliferation,
- difficulty in adjusting to the highly nonpolar conditions, which can cause microbial cells to rupture by dissolving the cytoplasm membrane lipids [20].

Mukherjee et al. (2014) showed that there was a decrease in microbial diversity and evenness in creosote-contaminated soils; however, an increase in total microbial activity was evident. This was likely due to the adaption and/or enrichment of the indigenous soil microflora with hydrocarbon-degrading capacities [20].

Although the vast majority of microorganisms at a given site will be unable to survive a hydrocarbon contamination event, there will be hydrocarbon-tolerant and hydrocarbon-degrading species that will survive and proliferate [1]. The exchange of genetic information, nutrients, or metabolites able to elicit gene activation can occur between microorganisms via a number of mechanisms. Broadly, these include indirect exclusive exchange (e.g., phagosome-mediated transfer), indirect transfer via diffusion of chemicals or direct transfer (e.g., contact-mediated transfer) of cellular content, and/or electron transfer. This transfer of metabolites, nutrients, and other cell–cell interactions may lead to enhanced hydrocarbon-degrading organism fitness or may select for parasites, which will reduce microbial diversity [21].

Khudur et al. (2015) observed changes in bacterial soil communities two weeks after being exposed to diesel at a concentration of 40 mL/kg [22]. A significant reduction in microbial diversity and evenness was observed. Following hydrocarbon contamination to a site, dynamic ecological balances are altered, and the site becomes dominated with a few select species [23]. However, the number of dominating species rapidly increases within 4 weeks following contamination [24], presumably due to the generation of many degradation intermediates and overall reduced toxicity.

Many changes in the characteristics of a microbial community occur during adaptation of a microbial community following a contamination event. This may include physiological and morphological changes in individual cells, along with changes in the ecological dynamics of the entire community [25].

Physiological characteristics (e.g., growth rates and innate resilience to stressors) can be a driver of adaptation. The diversity of microbial populations may result in favorable genomic variations, some of which may benefit the overall community [24]. Morphological characteristics include any changes in the shape and the size of the microbial cells and may take place to increase their transportation abilities in the soil matrix to reach the contaminants. Concentration and physio-chemical properties of specific contaminants are the major factors for any biological species to form adaptation mechanisms allowing them to use the pollutant as a substrate [26].

The degree of toxicity derived from hydrocarbon contamination on the biota can be influenced by many factors, including:

1. The hydrocarbon fraction itself: In saturated hydrocarbons, the chain length is highly correlated with direct toxicity. Fractions of lower molecular weight structures (C6 to C20) have been shown to be more toxic due to their high bioavailability; however, larger saturated chained hydrocarbons have increased mutagenic potential [5]. The toxicity of unsaturated hydrocarbon structures is not as predictable and may be influenced by reactive functional groups, water and membrane solubility, viscosity, and interactions of these compounds with the membrane and with membrane constituents.
2. The concentration of nutrients: the diversity of the microbial communities in soil with a higher concentration of N and P has been shown to increase the abundance and activity of the hydrocarbon-degrading microbial community [22].
3. Co-contamination with other chemicals (i.e., heavy metals): higher toxicity of petroleum hydrocarbon to soil microorganisms was reported with the presence of heavy metals as a co-contaminant when compared with the toxicity of petroleum hydrocarbon as the only contaminant [27]. Khudur et al. (2019) showed that hydrocarbon-contaminated soils were more recalcitrant to microbial bioremediation when co-contaminated with lead (Pb). Lead inhibits many metabolic pathways, such as the enzymatic and respiratory processes of many bacteria, and creates additional stress to hydrocarbon-degrading species [28].

Oil pollution events can have strong, immediate negative effects on the indigenous soil microbial community by reducing the diversity and total biomass [29]. However, the soil microbial community possesses an array of mechanisms of resilience. Bacteria with short life cycles can quickly employ coping strategies to these changes in the environment (i.e., production of secondary metabolites, sporulation, develop mutualistic interactions with other microbial species, etc.) [30]. Microbial biofilms in soil have natural resistance to perturbations as they are able to protect their constituents through slower diffusion rates and the co-metabolism of organic pollutants [31].

## 4. Physiochemical Factors Affecting Natural Attenuation of Petrogenic Hydrocarbons

The potential for natural attenuation of petroleum hydrocarbon-contaminated soil is not only affected by biotic factors, but also by abiotic factors such as salinity, moisture availability, nutrient availability, and soil chemistry. These abiotic factors are influenced by hydrocarbon contamination and significantly affect the capacity for microbial pollution catabolism.

### 4.1. Nutrients and Additives

For microbial utilization of oil to occur, access to nutrients is required [32]. Nutrients are often locked up by oils and become inaccessible to plants and microorganisms. Nutrients are required for generating the building blocks of new plant and microbial cells, along with supporting the proper functioning of all metabolic and structural processes of cells. In addition, hydrocarbon degradation may utilize certain nutrients as terminal electron acceptors for microbial catabolism pathways (common in anaerobic hydrocarbon degradation) [33].

Changes in nutrient levels also alter ecological relationships and interactions between microbial communities, such as communalism or parasitism relationships. Biostimulation, the application of nutrients has been shown to result in elevated hydrocarbon degradation; however, it has also been shown to select for pathogenic bacterial species which reduce the fitness of other hydrocarbon-degrading species. The addition of complex organic substrates like mature compost can cause desorption of oil from the soil due to its high humic content [34], leading to increased bioavailability of the oily compounds and higher bioremediation rates.

### 4.2. Salinity

High salt concentrations create a selective pressure which makes conditions unfavorable for many microbial species due to changes in osmotic pressure and the locking away of nutrients. “Salting out” occurs when salts reduce the solubility and availability of hydrophobic organic compounds [35]. Salinization of oil fields is a common occurrence [36] which may lead to a decrease in microbial respiration, and consequently a decrease in the rate of bioremediation for a site [37].

Some microbial consortias have been found to be robust and exhibit functional redundancy for the degradation of diesel from saline soils. Riis et al. observed that biodegradation of diesel in soil could be achieved if the salinity was below 15% (*w*/*w*) using consortia from the genera *Cellulomonas*, *Bacillus*, *Dietzia*, and *Halomonas*. [38]. Kleinsteuber et al. observed that microbial communities from naturally saline soils (6.4% *w*/*w*) exhibited functional redundancy for the degradation of diesel, with no significant decrease in activity with increases up to 20% (*w*/*w*) salinity [39]. A common halostress coping mechanism for bacteria is to “salt-in cytoplasm”, whereby the bacteria increase the concentration of potassium and chloride ions in their cell [40]. Biostimulation using K^+^ and Ca^2+^ ions help microorganisms buffer against osmotic stress and can help enhance their hydrocarbonoclastic activity in intertidal sediments [41].

Extreme salinity caused by oil field brine (200 dS m^1^) was shown to reduce the microbial oil utilization potential of soil contaminated with motor oil by 44% in clay loam soils, and 20% for sandy-clay loam soils [42].

### 4.3. Drought/Rainfall/Moisture

Water is not only necessary for the uptake of nutrients and the secretion of waste products within microbial cells, but is involved in practically all reactions within the cell. Water molecules aid in the stabilization of proteins, DNA, and lipids and in maintaining the structural integrity of microbial cells. Furthermore, water is required extracellularly to transport microbial-derived macromolecules for interactions with the environment. One such example is the transportation of genetic material from one microbial species to another via plasmids [43,44].

The lack of water negatively impacts soil microbial communities by hampering their interactions with each other and the environment, which may lead to a reduction in abundance, diversity, and structure [45]. This phenomenon was observed by [46], where after only 180 days of simulated drought conditions using semiarid agricultural soil from southern Spain, the activity of the soil microbial community substantially decreased, resulting in reduced C and N mineralization. These negative effects of drought were abated by the addition of compost to the soil at a rate of 100 t ha^−1^.

A combination of phytoremediation and microbial-driven bioremediation may be useful for the remediation of oil contamination under drought conditions. Phillips et al. observed that the microbial communities in the rhizosphere of Altai wild rye plants contained 100 times more endophytic hexadecane degraders than other plants under drought conditions [47]. How microbial communities respond to cycles of extreme desiccation and rewetting may have profound impacts on bioremediation. Small changes in community structure in response to wet/dry cycles may potentially hide large changes in the relative abundance of certain key microbial groups in soil communities [48]. The potential effects of drought on oil bioremediation are not well understood, but extrapolating from general microbial response studies, droughts are likely to reduce oil bioremediation rates [49].

Excess moisture may also impede bioremediation by saturating soil, reducing the capacity of O_2_ to dissolve into the water. This causes aerobic soils to become anaerobic [50]. Lahel et al. observed >20% decrease in remediation efficiency of diesel when moisture was increased from 10% to 30% in a series of microcosm experiments [51].

## 5. Microbe–Microbe Interactions

Microorganisms have a tremendous ability to modify their environment to provide ideal conditions for proliferation. A number of microbial relationships enable them to gain a competitive advantage within particular ecological niches and help the microbial community survive in adverse environments. These interactions include; parasitism, predation, competition, amensalism, commensalism, and mutualism [52].

Microbial communities present in natural ecosystems (i.e., bacteria and fungi (from different species)) proliferate together in a synergetic relationship and produce a remarkable cocktail of primary and secondary bioactive molecules (metabolites) including oxidative and hydrolytic enzymes. These natural products are essential for cell proliferation and have been implicated in the mineralization of varying hydrocarbon fractions [53]. Single-strained microbial cultures lack the multitude of hydrocarbon-degrading mechanisms which may be found within a consortium [54,55].

Mixed microbial community interactions have been shown to create conditions favorable for survival. One such example is the commensal relationship between cyanobacteria and other native bacterial species. Cyanobacterial mats were shown to increase the dissolved oxygen concentration in the zone around them through photosynthesis, creating aerobic pockets that were utilized by native bacteria which can degrade hydrocarbons [56]. Table 1 shows several different genes from different microorganisms which are associated with hydrocarbon degradation.

Mapping the activity and relationships between microorganisms in a complex community over time is a complicated task. However, its importance for generating well-functioning consortia for the specific site and contamination remediation is essential. Deng et al. utilized multiple network approaches to identify the co-occurrence (positive) or mutual exclusion (negative) interactions within microbial communities in groundwater. A positive relationship was more likely to be due to mutualism or commensalism, whereas negative relationships can be attributed to competition, predation, and amensalism [93].

## 6. Microbial Hydrocarbon Utilization

### 6.1. Aerobic Hydrocarbon Catabolism

In aerobic conditions, microbial hydrocarbon degradation is elevated when compared with anaerobic degradation. A driving factor influencing increased respiration in aerobic conditions is that there is no thermodynamically unfavorable requirement to introduce oxygen into the hydrocarbon via hydration [94].

Microbial aerobic catabolism of aliphatic hydrocarbons proceeds when the carbon backbone of the contaminant is cleaved, or functional substitution occurs via the loss of an electron to molecular oxygen [95]. These electron-carrier-dependent reactions are carried out by bacterial mono-oxygenase enzymes, converting the n-alkane hydrocarbon to their respective alcohol [96]. These hydroxylated products then enter peripheral metabolic pathways of the bacterial cell and are further oxidized, breaking apart C–C bonds in a step by step manner, resulting in smaller constituents that enter the primary metabolic pathway of the cell via β-oxidation [97].

Bacterial catabolism of aliphatic compounds has had been widely documented; however aromatic compounds require different microbial metabolic pathways for the attack. With increasing complexity comes increasing recalcitrance to degradation [98]. Low-molecular-weight PAHs (LMW PAHs) are generally more soluble, which increases their bioremediation potential. High-molecular-weight PAHs (HMW PAHs) may be too large to fit into the active site of many enzymes, and alternate degradation pathways may be necessary [58]. Furthermore, their decrease in water solubility and increase in potential carcinogenicity is counterproductive for microbial degradation [77].

Bacterial aerobic degradation of aromatics utilizes oxygen as a final electron acceptor and a substrate for hydroxylation and oxygenolytic ring cleavage reactions [99]. Quintessentially, bacterial aerobic PAH degradation involves the use of oxygenase enzymes (predominantly monooxygenases or dioxygenases). The aromatic ring is hydroxylated via oxygenase enzymes which form a cis-dihydrodiol, which then transforms to a diol intermediate via a dehydrogenase. The ortho-cleavage or meta-cleavage pathways then use oxygenase enzymes to destroy the aromatic ring and produce daughter products (i.e., catechols, which later transform to intermediates of the citric acid cycle) [58]. An alternative bacterial PAH-degrading pathway is the P450-mediated pathway, which can also be utilized by nonligninolytic fungi [77]. Several alternative pathways, which all involve the saturated ring cleavage via hydrolysis have been outlined previously [100].

### 6.2. Anaerobic Hydrocarbon Catabolism

The bacterial degradation of hydrocarbons in anaerobic conditions proceeds with metabolic pathways which employ alternative electron acceptors to oxygen (O_2_) (i.e., nitrate, metal ions, and sulphate, to name a few). The type of electron acceptor may vary among environmental site characteristics, different redox zones, and the microorganisms present within. Generally, the first alternative electron acceptor to be used by microorganisms for hydrocarbon catabolism following depletion in atmospheric oxygen is nitrate, as nitrate-reducing bacteria are often facultative anaerobes. Manganese, iron, and sulfate contain redox potentials below that of nitrate and are subsequently employed by their corresponding inorganic ion-reducing bacterial species. Methanogenesis possesses a lower redox potential than that of metal ions and sulfur and is dependent on by-products generated from fermentative and acetogenic bacterial species for supplying electron acceptors (i.e., H_2_/CO_2_, formate/acetate) [101,102].

Five dominant pathways have been identified for anaerobic bacterial hydrocarbon degradation; however, slight variations of these may also transpire. These anaerobic pathways include (1) addition of fumarate to the hydrocarbon chain, (2) hydroxylation of the hydrocarbon chain, (3) carboxylation of aromatics, (4) hydration of alkenes/alkynes, and (5) reverse methanogenesis [102].

#### 6.2.1. Addition of Fumarate to the Hydrocarbon Chain

The addition of fumarate to a hydrocarbon chain by anaerobic bacterial species results in a cascade of hydrocarbon chain rearrangements, which ultimately end up being processed by the β-oxidation pathway, resulting in the formation of alkylsuccinates. These alkylsuccinates can then be degraded by ligation to coenzyme A, carbon-skeleton re-arrangement, and beta-oxidation. Aliphatic and aromatic hydrocarbons can be degraded via this method [103].

Bian et al. (2015) reported the degradation of alkanes via fumarate addition in anaerobic oil reservoirs. This hydrocarbon-degrading mechanism proceeds with alkylsuccinate synthetase facilitating the binding of an n-alkane to the subterminal or terminal (with propane) carbon of fumarate. The resulting products are 2-(1-methylalkyl) succinates (or 2-alkylsuccinates). 2-(1-Methylalkyl) succinate is later rearranged to (2-methylalkyl)-malonyl-CoA, and decarboxylated to a 4-methylalkyl-CoA derivative which undergoes β-oxidation [104].

A number of nitrifying and sulfate-reducing bacteria (i.e., *Thauera aromatic* and *Desulfobacula toluolica*) are able to utilize toluene as a sole carbon source and break it down via the addition of fumarate to the carbon chain. In this process, benzylsuccinate synthase (BSS) facilitates the binding of toluene to the fumarate, forming (R)-benzylsuccinate, which is subsequently metabolized by the β-oxidation pathway [105].

Safinowski and Meckenstock et al. showed anaerobic degradation of the PAH, 2-methyl-naphthalene by a sulfate-reducing enrichment culture. Within this study, fumarate addition to the methyl group of 2-methylnaphthalene resulted in the formation of 2-napthylmethylsuccinic acid, which was subsequently processed by the β-oxidation pathway to form 2-naphthoic acid [106].

#### 6.2.2. Oxygen Independent Hydroxylation of the Hydrocarbon Chain

For the degradation of numerous ethyl- and propyl-substituted aromatic and heteroaromatic hydrocarbons, oxygen-independent hydroxylation of the hydrocarbon chain is common. Ethylbenzene dehydrogenase (EBDH) catalyzes the hydroxylation of the hydrocarbon chains next to the ring with water (as opposed to O_2_ in aerobic systems) and converts ring-substituted mono- and bicyclic aromatic compounds to alcohols (e.g., the conversion of ethylbenzene to (S)-1-phenylethanol) [107]. Following this, subsequent enzymatic dehydrogenase, carboxylase, and ligase reactions occur before the structure is thiolytically cleaved into benzoyl-CoA and acetyl-CoA [107].

#### 6.2.3. Carboxylation of Aromatics

Although the absolute certainty of carboxylation as a hydrocarbon-activating mechanism needs further reinforcement in the literature, identification of aromatic hydrocarbon-derived carboxylic acids with CO_2_-derived carboxyl groups provides evidence for the activation of hydrocarbons via carboxylation [108].

#### 6.2.4. Hydration of Alkenes/Alkynes

Alkene and alkyne hydrocarbons in anaerobic conditions may be degraded via the addition of a water molecule to the unsaturated double or triple bond, converting the parent hydrocarbons to alcohols, ketones, or aldehydes [109]. Rontani et al. utilized bacterial consortia from marine sediments to degrade squalene and suggested an anaerobic degradation pathway where the double bonds were hydrated, resulting in tertiary alcohols and ketones being formed [110].

#### 6.2.5. Reverse Methanogenesis

Microbial decomposition of methane is predominantly facilitated via reverse methanogenesis by *archaea,* although exceptions to this occur. Ettwig et al. showed degradation of methane in anaerobic conditions in the absence of *Archaea* and reported that the bacterial consortium coupled anaerobic oxidation of methane to denitrification [111].

Anaerobic methane oxidizers degrade methane using a number of different terminal electron acceptors (i.e., nitrate/nitrite, sulfate, iron, and manganese). The exact pathways for reverse methanogenesis have not been fully elucidated as the process is expected to differ between the electron acceptors utilized. The reaction usually proceeds slowly and is dependent on syntrophic/symbiotic interactions between microbial consortia which are difficult or unculturable in laboratory conditions [112].

Anaerobic methanotrophs from the domain archaea are thought to use the reverse reaction of methyl-coenzyme M reductase (the key enzyme in methanogenesis) for activation of methane. However, the bacterium *Methylomirabilis oxyfera* has the capacity to convert NO from reduced nitrite to N_2_ and O_2_, which makes it possible for methane monooxygenases to attack methane [113].

### 6.3. Syntrophy

**Figure 2:**
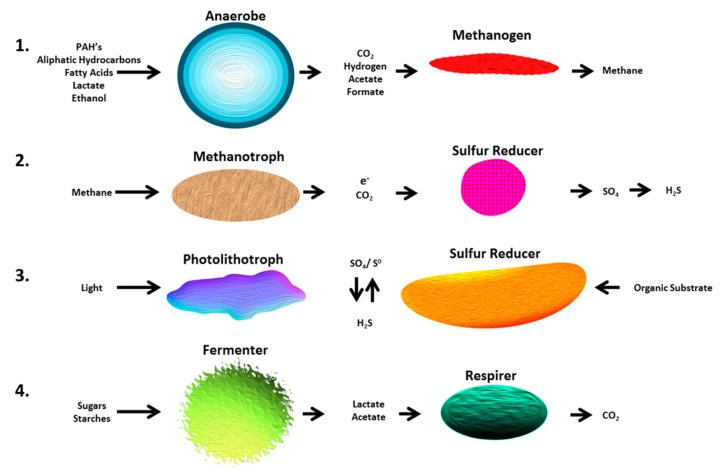
Syntrophic interactions: by-products from one type of microorganism aid in the growth and proliferation of another (adapted from Wintermute & Silver, 2010, permission has been given by CSHL Press). PAH: polyaromatic hydrocarbon.

Syntrophy is a process whereby the intermediate or end products from one species metabolism are utilized by another to facilitate proliferation (Figure 2) [114]. For microbial anaerobic hydrocarbon degradation, it is common for fermentative, syntrophic, acetogenic, and methanogenic bacteria to form symbiotic relationships [115]. These interactions help to facilitate the progression of otherwise energetically unfavorable reactions (degradation of hydrocarbons via different metabolic pathways) [116].

Within an anoxic hydrocarbon-contaminated site, hydrocarbons, proteins, and polysaccharides may be destroyed by hydrolytic bacteria, forming amino acids, sugars, starches, and fatty acids. These breakdown products can then be utilized by fermentative bacterial species which produce acetate, formate, propionate, butyrate, lactate, carbon dioxide, and hydrogen gas [114] (Figure 2). The resulting conditions are ideal for methanogens to thrive, and they utilize these substrates to generate methane [117]. Reverse methanogens (e.g., methanotrophic bacteria (type II) [117]) can convert methane to carbon dioxide and produce free electrons, which sulfur-reducing bacteria can then utilize to generate hydrogen gas (in sulfate-devoid conditions) [118]. The evolution of hydrogen gas further supports the proliferation of methanogenic species, providing a feedback loop [114].

The degradation of alkanes in anaerobic conditions has also been reported with the different electron acceptors and methanogenesis. A review by Mbadinga et al. compiled information on the processes and consortia involved in the degradation of these hydrocarbons [119]. Fumarate addition and carboxylation were proposed as the main mechanisms for the initial activation of the alkanes in anaerobic conditions, and the former was confirmed as the exclusive mechanism under sulfate-reducing conditions [120].

Anaerobic degradation of alkylbenzenes coupled to nitrate reduction with symbiotic associations has been observed in an enrichment dominated by *Geobacter* spp. with annamox bacteria (*Plantomycetales*) when growing on cyclohexane as the only carbon source [121].

The degradation of alkanes in methanogenic conditions has been reported under two main degradation pathways. Initially, the hydrocarbon is decomposed to acetate and H_2_ by syntrophic bacteria; following this, methane can be formed by the acetoclastic methanogens such as *Methanosatea concilii,* while the second pathway includes another group of archaea, the hydrogenotrophic methanogens (*Methanospirillum* and *Methanoculleus*) that convert CO_2_ and H_2_ to methane.

The anaerobic degradation of BTEX has been studied in several aquifers around the world [122], and some degradation mechanisms have been proposed for benzene [123,124]. Nitrate-, sulfate-, and iron-reducing conditions often result in methanogenesis. Thus, the utilization of hydrocarbons in anoxic conditions has been shown to be carried out by phylogenetically diverse microorganisms. The dominant interactions between these species tend to be syntrophic, with carbon and electron sharing occurring during the degradation pathway.

The taxa involved in the degradation of hydrocarbons has been listed previously [122], and studies dealing with methanogenic hydrocarbon-degrading cultures and their syntrophic processes have been compiled by Gieg and collaborators [125]. Some of the microorganisms involved in the initial attack of hydrocarbons belong to the *Pelotomaculum*, *Clostridium* (within the *Peptococcaceae*) genus, and the *Deltaproteobacteria*. Some of the *Synthropus* include the *Syntrophoceae* and *Smithella* and have been found in multiple contaminated sites [126,127]. The final stages of hydrocarbon degradation in anaerobic conditions are often performed by acetoclastic methanogens such as *Methanosaeta concilii* or hydrogenotrophic methanogens such as members of the *Methanoculleus* within the *Methanobacteriaceae* [125,128,129].

### 6.4. Microbial Interactions That Can Indirectly Aid Hydrocarbon Degradation

Bacteria–bacteria, bacteria–fungi, and bacteria–fungi–plant interactions are ubiquitous in nature and are responsible for a vast array of differing environmental coping mechanisms. Signaling between bacterial species can be both dependent and independent of physical contact. Gram-negative cells tend to secrete low-molecular-weight pheromones (i.e., N-acyl homoserine lactones (AHLs)), which can regulate antibiotic production, cell differentiation, genetic exchange, cell aggregation and biofilm production, protein secretion and so on. [130].

Gram-positive bacteria tend to use small oligopeptides and proteins to transmit information to one another. Oligopeptides usually transfer through the cell membrane and dock to extracellular two-component adaptive-response proteins. These proteins act as receptors and transmit signals for genetic regulation, conjugation, sporulation, virulence, and so on. [131]. A number of microbial species have also adapted both mechanisms as an evolutionary proliferation strategy (i.e., *Vibrio harveyi)* [132].

Biofilm-producing microbial species are often found within extracellular polymeric substances (EPS). In addition, EPS have been shown to concentrate BTEX pollutants, removing them from soil or water matrices [26]. This barrier can harbor individual species or a number of interacting species and can protect against varying environmental extremes, sheer forces, dehydration, antibiotics, acid and UV damage, predators, and high concentrations of toxic chemicals and pollutants. Biofilms have a strong ability to transfer information via cell–cell contact mechanisms and also through intercellular interactions via chemical signals (i.e., quorum sensing). In some bacterial biofilms (i.e., *Alcaligenes* sp. strain d2), air-containing cavities are present which facilitate the growth of microcolonies. This phenomenon enables the transportation and removal of biomolecules and oxygen to different members of the community [132].

Oil degradation by microbial interactions can be facilitated by a number of indirect processes. The production of biosurfactants is a common mechanism for bacteria to solubilize viscous oils [133]. This transforms them into a form which is more bioavailable for utilization [26]. Bacteria may also secrete biomolecules that facilitate functional gene expression for hydrocarbon tolerance/degradation reduction in predators through antibiotic production, glucose production to stimulate population growth amongst hydrocarbon degraders, heavy metal chelator production which reduces metals toxicity, extracellular enzymes which may attack recalcitrant hydrocarbons, and so on. [134,135]. In addition, microbial species can secrete molecules that can interact with fungi and plants. One such example is the production of 1-aminocyclopropane-1-carboxylate (ACC) deaminase by a rhizobia bacterium. This molecule alters the plants ethylene production and in turn induces plant tolerance to environmental stresses [136].

## 7. Conclusions and Future Directions

Petroleum hydrocarbons are destructive to ecosystem health, economic health, and human health. Remediation of these sites is therefore necessary to restore functionality, whether that is for environmental preservation or urban development.

Technologies currently used to remove petroleum hydrocarbon-contaminated environments are costly and are often conducted off site, which requires the initial physical removal of contaminated soil. In situ technologies all have their inherent downfalls including high cost (soil flushing), suitability only for specific soil types (soil vapour extraction), inability to remediate all zones of a contaminated site (soil vapour extraction), site may not be used for development following remediation (solidification/stabilization), formation of undesirable by-products (electrokinetic remediation), potential for process-induced detrimental effects (chemical oxidation), and inefficient degradation times (bioremediation).

Natural attenuation does not possess the capacity to remediate highly petrochemical contaminated sites at appreciable rates. As a result, alternative technologies such as bioremediation, which is environmentally friendly and cheap compared with conventional treatment approaches, have been developed.

The application of bioremediation techniques has in the past been limited by the knowledge base of environmental science and microbiology. For the site-specific application of bioremediation, the physical and chemical parameters have been the better-understood aspects, thus the basis for models and assessments in the past.

Understanding the complexity of microbial consortium dynamics in terms of degradation of recalcitrant hydrocarbon mixtures has only recently become practicable with the use of affordable/economically viable high-throughput techniques (i.e., next generation sequencing (NGS) and microarray technologies). This area of community interaction analysis for complex environmental systems (i.e., contaminated soil) is still in its infancy; however, without understanding the functional and structural role of microorganisms within whole communities, developing remediation strategies will remain limited.

## Figures and Tables

**Table 1:** Microbial genes implicated in hydrocarbon degradation with their function/pathway and representative host species.

Genes	Function	Example Organism of Origin	Gene Location (Chromosome or Plasmid)	Reference
*Nah* genes (G, T, H, I, N, L, O, M, K, J, Y, W)	Naphthalene catabolic genes (nah Y is chemotaxis gene and nah W may aid in adapting to extreme conditions)	*Pseudomonas putida G7*	Chromosome and/or plasmid	[57]
*Ndo* genes	Naphthalene dioxygenases	*Pseudomonas putida*	Chromosome and/or plasmid	[58,59]
*Dox* genes (bphA bphE bphF bphG)	Dibenzothiophene oxidation (meta cleavage pathway)	*Pseudomonas. sp.C18*	Plasmid	[58,59,60]
*Pah*	Naphthalene-Phenanthrene dioxygenase genes	*Pseudomonas putida OUS82, Pseudomonas aeruginosa Pak1*	Plasmid	[58]
*Nag* gene	Naphthalene dioxygenase genes	*Ralstonia sp. strain U2*	Plasmid	[61]
*Phn* genes (C, I, H)	Phenanthrene degradation genes	*Alcaligenes faecalis AFK2 strain*	Plasmid	[62]
*Fln* genes (RB, ED1)	Fluorine degradation	*Terrabacter sp. strain DBF63*	Plasmid	[63]
*Pht* genes (Aa, Ab, B, Ac, Ad)	Degrading polycyclic aromatic hydrocarbons (PAHs) to ring cleavage metabolites (phthalate)	*Mycobacterium vanbaalenii PYR-1*	Plasmid	[64]
*CAT* genes (A, B, C, R)	Catechol catabolic genes, cat central ortho pathway	*Burkholderia strain LB400*	Chromosome	[65,66]
*Pca* genes (ligA, fldV, pmdA, proOa, (ligB, fldU, pmdB, pmdC, proD, pcmB,	Catabolism of the phenolic compounds (protocatechuate)	*Agrobacterium tumefaciens*	Chromosome	[67]
*Gdo* genes	Lignin and salicylate degradation (bacterial and fungal), gentisate 1,2-dioxygenase, cleavage of the gentisate aromatic ring	*Rhodovulum sp. Strain NI22, Pseudomonas alcaligenes NCIMB 9867 (strain P25X)*	Chromosome and/or plasmid	[68,69,70]
*Alk* genes (B, F, G, H, J, K, L)	Hydroxylation of aliphatic hydrocarbons	*Pseudomonas oleovorans*	Chromosome and/or plasmid	[71,72]
*Ben* genes (A, B, C, D)	Benzoate catabolic	*Halomonas organivorans*	Chromosome	[71]
*CYP* genes (*pb*-1, *pb*-2 and *pb*-3, CYP153)	Fungal and Bacterial Cytochrome P450 monooxgenase	*Basidiomycetes, Acinetobacter* calcoaceticus EB104	Chromosome	[73]
*LadA*	Long-chain alkane mooxygenase-Degradation of long-chained alkanes	*Geobacillus thermodenitrificans NG80-2*	Plasmid	[71]
*Alm* (A)	A flavin-binding mooxygenase-Degradation of long-chained alkanes	*Acinetobacter sp. DSM 17874*	Chromosome	[71]
*Phd* genes (E, F, G, H, I, J, K)	Aromatic hydrocarbon degradation	*Comamonas testosteroni GZ39*	Chromosome	[62,74]
*Nid* genes (A, A3, B2, B3, D,)	Pyrene degradation	*Mycobacterium sp. PYR-1*	Chromosome	[58]
*TOL* genes (xyl)	Toluene degradation	*Pseudomonas putida mt-2*	Plasmid	[75]
*Alm*	N-Alkanes (C32 and beyond)	*Acinetobacter sp. DSM 17874*	Chromosome	[76]
*Phn* genes (B, C, R, S)	PAH degradation	*Burkholderia sp. RP007*	Plasmid	[57]
*Nar* genes (Aa, Ab, B)	Naphthalene degradation	*Rhodococcus sp. NCIMB12038*	Plasmid	[77]
*Nid* (A)	High-molecular-weight pahs degradation (e.g., pyrene and Fluoranthene)	*Mycobacterium sp. PYR-1*,	Chromosome	[78]
*Dbf* genes (A1, A2)	Terminal oxygenase genes of angular dioxygenase (Fluorine degradation)	*Terrabacter sp. strain DBF63*	Plasmid	[63]
*Xyl* genes (X, Y, Z, L)	M-xylene degradation	*Pseudomonas putida mt2*	Plasmid	[79,80]
*Tod* genes (A, B, C1, C2, D, E, C1C2BA, C1C2BAD, CIC2BADE)	Toluene dioxygenase (tod) metabolism of toluene, benzene, and ethylbenzene.	*Pseudomonas putida*	Chromosome and/or plasmid	[81,82]
*tom*	Toluene ortho-monooxygenase, oxidation of the polycyclic Aromatic hydrocarbons naphthalene and fluorene	*Pseudomonas mendocina KR1*	Plasmid	[83]
*Tbu* genes (E, F, G, K, I, H, J)	BTEX, meta cleavage	*Pseudomonas aeruginosa PAO1, R.*	Chromosome	[84]
*tmo*	Multicomponent monooxygenase Enzyme complexes involved in aerobic benzene, toluene, ethylbenzene and xylene (BTEX) degradation	*Ralstonia pickettii PKO1*	Chromosome	[85]
*Xyl* (E)	Catechol 2,3-dioxygenase (ring cleavage reaction in PAH degradation)	*Sphingomonas yanoikuyae*	Chromosome	[86]
*Dmp* genes (K, L, M, N, O, P, Q, B, C, D, E, H, F, G, I)	Involved in the meta cleavage pathway	*Pseudomonas sp. CF600*	Plasmid	[87,88]
*LiP* genes (A, D, LipJ	Fungal Lignin peroxidase	*Phanerochaete chrysosporium*	Chromosome	[89,90]
*MnP*	Fungal Manganese Peroxidase	*Phanerochaete chrysosporium*	Chromosome	[89]
*POD* genes	Fungal Lignin degradation (heme – peroxidase encoding)	*Bjerkandera adusta, Ganoderma sp.*	Chromosome	[91,92]
*HTP*	Fungal Lignin degradation heme-thiolate peroxidase (heme – peroxidase encoding)	*Rhodonia placenta*	Chromosome	[92]
*DyP*	Fungal Lignin degradation Dye-decolorizing peroxidase (heme – peroxidase encoding)	*Thanatephorus cucumeris*	Chromosome	[92]
